# Mitochondrial DNA copy number in peripheral blood leukocytes and the aggressiveness of localized prostate cancer

**DOI:** 10.18632/oncotarget.5889

**Published:** 2015-10-26

**Authors:** Huakang Tu, Jian Gu, Qing H. Meng, Jeri Kim, John W. Davis, Yonggang He, Elizabeth A. Wagar, Timothy C. Thompson, Christopher J. Logothetis, Xifeng Wu

**Affiliations:** ^1^ Department of Epidemiology, The University of Texas MD Anderson Cancer Center, Houston, Texas, USA; ^2^ Department of Laboratory Medicine, The University of Texas MD Anderson Cancer Center, Houston, Texas, USA; ^3^ Department of Genitourinary Medical Oncology, The University of Texas MD Anderson Cancer Center, Houston, Texas, USA; ^4^ Department of Urology, The University of Texas MD Anderson Cancer Center, Houston, Texas, USA; ^5^ Department of Surgery, Ruijin Hospital, Shanghai Jiao Tong University School of Medicine, Shanghai, China

**Keywords:** mitochondrial DNA copy number, prostate cancer, aggressiveness, recurrence, progression

## Abstract

We investigated whether low mitochondrial DNA copy number (mtDNAcn) in peripheral blood leukocytes at diagnosis was associated with an increased risk of the aggressive form of the tumor and disease progression among localized prostate cancer (PCa) patients. We recruited 1,751 non-Hispanic white men with previously untreated PCa from The University of Texas MD Anderson Cancer Center. mtDNAcn was categorized into three groups according to tertiles. We used multivariate logistic regression to estimate the odds ratios (ORs) and 95 percent confidence intervals (95% CIs) for the association of mtDNAcn with the risk of having aggressive PCa at diagnosis. We used Cox proportional hazards model to estimate hazard ratios (HRs) and 95% CIs for disease progression. We observed an inverse association between aggressiveness of PCa and mtDNAcn (*P* < 0.001). In multivariate analysis, compared to patients in the highest tertile of mtDNAcn, those in the second and lowest tertiles had significantly increased risks of presenting with the high-risk form of PCa, as defined by the D'Amico criteria, with ORs of 1.33 (95% CI, 0.89–1.98; *P* = 0.17) and 1.53 (95% CI, 1.02–2.30; *P* = 0.04), respectively. Furthermore, PCa patients in the lowest and second tertiles combined relative to those in the highest tertile had a 56% increased risk of disease progression (HR, 1.56; 95% CI, 0.96–2.54; *P* = 0.07). In summary, our results suggested that low mtDNAcn in peripheral blood leukocytes was associated with aggressive PCa at diagnosis and might further predict poor progression-free survival among localized PCa patients.

## INTRODUCTION

Mitochondria are key organelles that perform multiple central cellular functions such as energy production, cell proliferation, and apoptosis [1, 2]. Besides the nuclei, mitochondria are the only organelles in human cells that possess their own DNA (mtDNA). Each mitochondrion contains 1–15 mtDNA molecules [3, 4], and the number of mitochondria per cell ranges from several hundred to greater than 10, 000 mainly depending on the energy demand of the cell as well as environmental factors [5, 6]. In comparison to nuclear DNA, the mutation rate of mtDNA is about 10-fold higher due to the lack of introns and protective histones, limited DNA repair mechanisms, and its close proximity to the electron transport chain which generates reactive oxygen species [3, 7–10]. Alterations in mtDNA including mutations and changes in copy number have been associated with multiple cancers including prostate cancer (PCa) [11–18].

PCa, the most prevalent cancer among men in the US, accounts for 26% of all cancer cases in men [19]. When diagnosed at an early stage (i.e., localized or regional) the 5-year survival rate is almost 100%, but when the tumor has metastasized, the rate is only 28.0% [20]. The blood prostate-specific antigen (PSA) test is the most commonly used screening test for PCa. However, the PSA test often captures asymptomatic indolent tumors that have little or no lethal potential, thus causing over-diagnosis and over-treatment [19, 21]. In order to distinguish between indolent and aggressive PCa, multiple pre-treatment risk stratification systems have been proposed [22]. The most commonly used one is the D'Amico risk criteria, which groups localized PCa patients into three categories: low-risk, intermediate-risk, and high-risk based on total Gleason score, clinical stage and PSA levels at diagnosis [23]. However, these groups are still heterogeneous and do not explain all the variations seen in prognosis, treatment and management [22]; therefore, new markers for disease aggressiveness and progression are needed.

Multiple *in vitro* cell line studies showed that low mtDNA copy number (mtDNAcn) was associated with aggressive phenotypes of PCa [24–26], suggesting that mtDNAcn could be a potential biomarker for distinguishing indolent from aggressive PCa. However, evidence from human studies is very limited. Three previous human population studies on mtDNAcn and aggressive features of PCa at diagnosis produced conflicting results [26–28]. Regarding disease progression, to the best of our knowledge, no population study has prospectively investigated whether mtDNAcn at diagnosis is associated with disease progression of PCa.

In this study, we investigated whether low mtDNAcn in peripheral blood leukocytes was associated with aggressive features of PCa in a large cohort of patients with localized PCa. Furthermore, we investigated whether low mtDNAcn could predict disease progression independent of the D'Amico risk stratification system.

## RESULTS

### mtDNA copy number in peripheral blood leukocytes by selected characteristics of the study population

The distribution of selected characteristics of the study participants and mtDNAcn levels by selected characteristics are shown Table 1. The median follow-up time was 44.3 (range: 0.3–156.8) months. The majority of the participants were between 55 and 65 years old, overweight or obese, non-smokers or former smokers, had D'Amico intermediate-risk form of tumor, had a total Gleason score of 7, had T1 stage tumor, and had PSA at diagnosis <10 ng/ml. Radical prostatectomy (51.7%) was the most common treatment. mtDNAcn was significantly lower among patients who were older (*P* < 0.001) or obese (*P* = 0.04). Non-smokers seemed to have lower mtDNAcn but the difference was not statistically significant (*P* = 0.74). mtDNAcn decreased as the aggressiveness (defined by the D'Amico risk groups) increased (*P* < 0.001). In addition, mtDNAcn decreased as total Gleason score increased (*P* = 0.009), as tumor stage increased (*P* = 0.08), and as PSA levels at diagnosis increased (*P* = 0.21). mtDNAcn did not differ significantly among patients receiving different initial primary treatments (*P* = 0.54).

**Table 1:** mtDNAcn in peripheral blood leukocytes by selected characteristics of the study patients with localized prostate cancer

Characteristics^a^	Total (*N* = 1,751)	mtDNAcn^b^	*P* value^c^
**Age at diagnosis, years**			
<55	339 (19.4)	0.87 (0.37)	
55–65	836 (47.7)	0.81 (0.36)	
>65	576 (32.9)	0.75 (0.33)	<0.001
**BMI at diagnosis, kg/m^2^**			
<25	256 (17.6)	0.79 (0.33)	
25–29.99 (overweight)	674 (46.3)	0.81 (0.36)	
≥30 (obese)	527 (36.2)	0.76 (0.30)	0.04
**Smoking status at diagnosis**			
Non-smoker	819 (47.0)	0.80 (0.35)	
Former smoker	782 (44.9)	0.81 (0.35)	
Current smoker	140 (8.0)	0.82 (0.38)	0.74
**D'Amico risk group**			
Low	589 (33.6)	0.83 (0.38)	
Intermediate	830 (47.4)	0.81 (0.34)	
High	332 (19.0)	0.74 (0.31)	<0.001
**Total Gleason score**			
≤6	647 (37.0)	0.83 (0.37)	
7	881 (50.3)	0.80 (0.34)	
≥8	222 (12.7)	0.74 (0.31)	0.009
**Clinical tumor stage**			
T1	1,095 (62.5)	0.82 (0.36)	
T2	569 (32.5)	0.79 (0.35)	
T3–T4	74 (4.2)	0.74 (0.25)	0.08
**PSA at diagnosis**			
<10 ng/ml	1,527 (87.4)	0.81 (0.36)	
10–20 ng/ml	154 (8.8)	0.77 (0.29)	
>20 ng/ml	67 (3.8)	0.75 (0.31)	0.21
**Initial primary treatment**			
Radical prostatectomy	906 (51.7)	0.82 (0.36)	
Radiotherapy	376 (21.5)	0.79 (0.34)	
Surveillance or unknown^d^	427 (24.4)	0.80 (0.36)	
Other treatment^e^	42 (2.4)	0.80 (0.36)	0.55

a Reported as count (percentage)

b Reported as mean (standard deviation)

c For the differences in mtDNAcn by selected characteristics using ANOVA

d Patients undergoing active surveillance/watchful waiting or whose initial treatment information was unavailable

e Chemotherapy, cryoablation, high-intensity focused ultrasound, or transurethral resection of prostate

Abbreviations: mtDNAcn, mitochondrial DNA copy number; BMI, body mass index; PSA, prostate-specific antigen.

### mtDNA copy number and aggressiveness of localized prostate cancer

**Figure 1:**
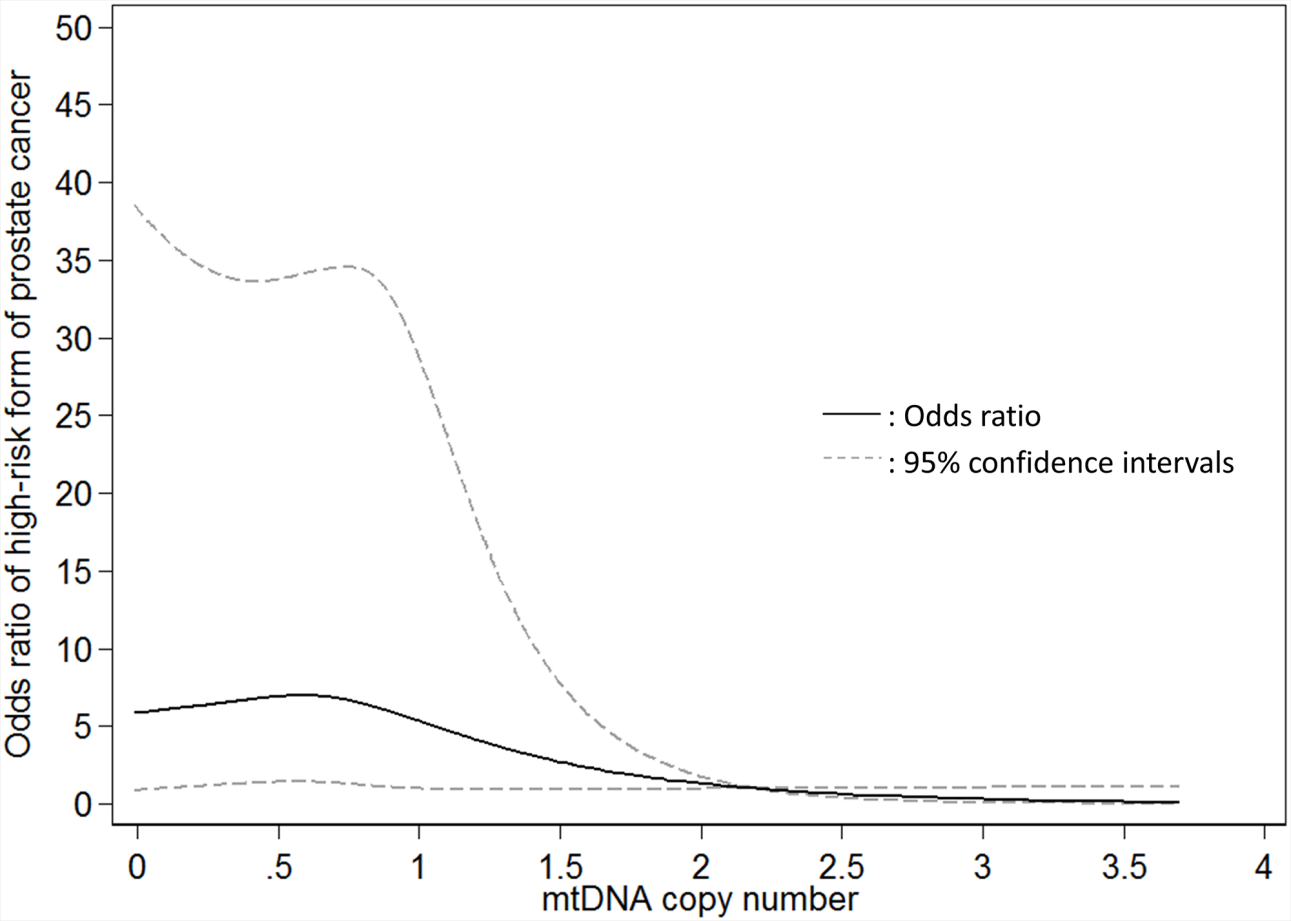
Restricted cubic spline modelling for the association of mitochondrial DNA copy number in peripheral blood leukocytes with high-risk form of localized prostate cancer in comparison to low-risk form of prostate cancer Four knots were used and the 99th percentile (2.195) was chose as the reference level.

The associations of mtDNAcn with the risk of presenting with the intermediate- and high-risk form of PCa are shown in Table 2. In univariate analysis, compared to patients in the highest tertile of mtDNAcn group, the ORs of presenting with high-risk form of PCa for patients in the second and lowest tertiles were 1.44 (95% CI, 1.03–2.03; *P* = 0.04) and 1.96 (95% CI, 1.40–2.75; *P* < 0.001), respectively; the associations were attenuated but remained statistically significant after adjustment of age, BMI, smoking status and pack-year (second *vs*. highest tertile: OR, 1.33; 95% CI, 0.89–1.98; *p* = 0.17; lowest *vs*. highest tertile: OR, 1.53; 95% CI, 1.02–2.30; *P* = 0.04; *P* for trend = 0.047). When we conducted stratified analysis by age (<60 *vs*. ≥60 years), BMI (<30 vs. ≥30 kg/m^2^), and smoking status (never-smokers *vs*. ever-smokers), the results were similar (Supplementary Table 1). Meanwhile, mtDNAcn was not significantly associated with the intermediate-risk form of PCa. In addition, we used the restricted cubic spline to model the potential non-linear association of mtDNAcn with the high-risk form of PCa. As shown in Figure 1, a lower mtDNAcn was associated with a higher risk of presenting with the high-risk form of PCa, but the effect seems to plateau at mtDNAcn of 0.75.

**Table 2:** mtDNAcn in peripheral blood leukocytes and aggressiveness of localized prostate cancer at diagnosis

mtDNAcn	Low-riskform of PCa	Intermediate-riskform of PCa			High-riskform of Pca		
	*N* (%)	*N* (%)	Adjusted OR^a^ (95% CI)	*P* value	*N* (%)	Adjusted OR^a^ (95% CI)	*P* value
3^rd^ tertile (highest)	214 (36.3)	285 (34.3)	Reference	N/A	84 (25.3)	Reference	N/A
2^nd^ tertile	201 (34.1)	269 (32.4)	0.89 (0.66–1.19)	0.42	114 (34.3)	1.33 (0.89–1.98)	0.17
1^st^ tertile (lowest)	174 (29.5)	276 (33.3)	1.05 (0.78–1.42)	0.75	134 (40.4)	1.53 (1.02–2.30)	0.04
*P* for trend			0.77			0.047	

a Adjusted for age, BMI, smoking status and pack-year

Abbreviations: mtDNAcn, mitochondrial DNA copy number; PCa, prostate cancer; OR, odds ratio; CI, confidence interval

We further investigated whether mtDNAcn was associated with Gleason score overall and by PSA levels (Supplementary Table 2). Overall, low mtDNAcn was borderline significantly associated with a Gleason score ≥8 (lowest *vs*. highest tertile: OR, 1.53; 95% CI, 0.97–2.43; *P* = 0.07), and the association was only present among patients with PSA ≤4 ng/ml (OR, 4.88; 95% CI, 1.30–18.39; *P* = 0.02).

### mtDNA copy number and disease progression of localized prostate cancer

**Figure 2:**
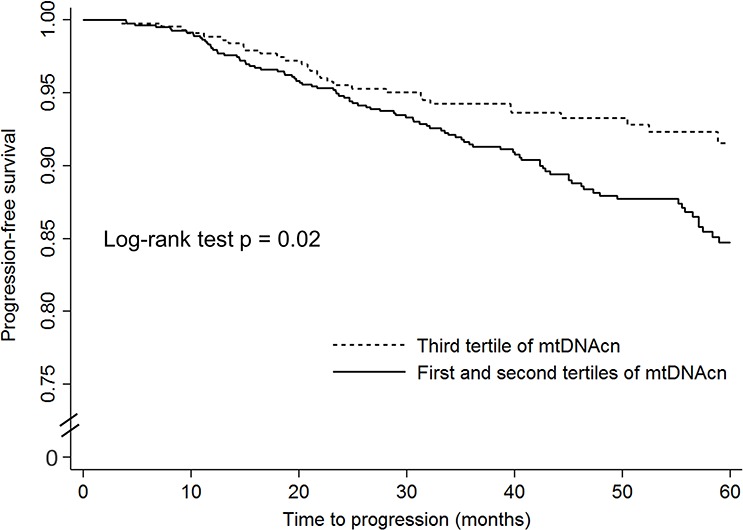
The Kaplan–Meier progression-free survival curves by mitochondrial DNA copy number in peripheral blood leukocytes among localized prostate cancer patients who received active treatments

The unadjusted and adjusted associations of mtDNAcn with disease progression among patients who received active treatments are shown in Figure 2 and Table 3. Since the rate of disease progression is relatively low among patients with localized PCa, we combined the lowest and second tertiles for the analysis of disease progression to increase statistical power. As shown in Figure 2, compared to patients in the highest tertile, patients in the first and second tertiles combined had worse progression-free survival (*P* = 0.02). In crude Cox regression analysis (Table 3), compared to PCa patients in the highest tertile of mtDNAcn group, those in the second and lowest tertiles combined had an increased risk of disease progression with a HR of 1.59 (95% CI, 1.09–2.34; *P* = 0.02); after adjustment of age, BMI, smoking status, and pack-year, D'Amico risk groups, and treatments, the multivariate-adjusted HR was similar and borderline significant (HR, 1.56; 95% CI, 0.96–2.54; *P* = 0.07). We also conducted stratified analysis by age, BMI, smoking status, and D'Amico risk groups (Supplementary Table 3), and there was no evidence of heterogeneity between different strata.

**Table 3:** mtDNAcn in peripheral blood leukocytes and disease progression among localized prostate cancer patients who received active treatments

mtDNAcn	Progression *N* (%)	No Progression *N* (%)	Crude HR (95% CI)	*P* value	Adjusted HR^a^ (95% CI)	*P* value
3^rd^ tertile (highest)	35 (8.0)	401 (92.0)	Reference	N/A	Reference	N/A
2^nd^ and 1^st^ tertile	104 (12.5)	726 (87.5)	1.59 (1.09–2.34)	0.02	1.56 (0.96–2.54)	0.07

a Adjusted for age, BMI, smoking status, pack-year, D'Amico risk groups, and initial primary treatment

Abbreviations: mtDNAcn, mitochondrial DNA copy number; HR, hazard ratio; CI, confidence interval

## DISCUSSION

In this study of 1,751 patients with localized PCa, we found that low mtDNAcn in peripheral blood leukocytes was associated with aggressive features of PCa at diagnosis including higher D’ Amico risk groups and higher total Gleason score; in addition, our results suggested that low mtDNAcn might be associated with an increased risk of disease progression independent of the D’ Amico risk stratification groups.

Our findings are consistent with previous cell line studies which showed that low mtDNAcn was associated with aggressive phenotype of PCa cells [24–26]. Also, our study is consistent with human studies on other cancers including gastric [29], ovarian [30], renal [31], and breast [32] cancers, which showed that low mtDNAcn in tumor tissue was associated with aggressive features of the tumor. Three previous human studies on mtDNAcn and aggressive features of PCa reported inconsistent results [26–28]. The first study (*n* = 14) found that low mtDNAcn in PCa tumor tissue was associated with high-grade tumors (Gleason grade 8 and 9) [26]. The second study (*n* = 38) showed mtDNAcn in PCa tumor tissue or peripheral blood leukocytes had no significant association with tumor stage, Gleason score, and PSA levels [27]. The third study (*n* = 193) found that higher mtDNAcn in peripheral blood leukocytes was associated with more advanced tumor stage, higher Gleason score, and higher PSA levels [28]. The reasons for the inconsistency include random errors due to the small sample sizes of these prior studies, variations in the protocols for assigning Gleason score and tumor stage, variations in the measurements of PSA levels and mtDNAcn, and diverse genetic background of the study populations (e.g., the second study used a mixture of Caucasians and African Americans and the third study used a Chinese population from two hospitals).

Our study provided the first evidence that low mtDNAcn in peripheral blood leukocytes might be associated with an increased risk of disease progression of localized PCa, although after adjustment of age, BMI, smoking status, pack-year, D'Amico risk groups, and treatments, the associations were borderline significant, likely due to the relatively small number of events among localized PCa. Consistent with our study, a previous report among breast cancer patients found that reduced mtDNAcn in tumor tissues was associated with increased risks of disease progression and all-cause mortality [32]. On the other hand, Grupp *et al*. [33] analyzed the expression of MTC02, a 60 kDa non-glycosylated protein component of mitochondria, in the tumor tissues of PCa patients via tissue microarray, and found that high mitochondria content, as indicated by increased expression of MTC02, was associated with an elevated risk of biochemical recurrence. It should be pointed out that our study and Grupp's study used different measures (i.e., mtDNAcn *vs*. MTC02 expression) and therefore this discrepant result should be interpreted with caution.

Our findings are biologically plausible for several reasons. First, a decrease in mtDNAcn leads to insufficient oxidative phosphorylation and greater generation of adenosine triphosphate (ATP) by glycolysis [34]. These changes could result in a stronger tolerance to hypoxia and reduced dependence on mitochondrial oxidative phosphorylation, thus conferring growth advantage to tumor cells [26, 31, 35]. Second, a decrease in mtDNAcn may promote cells to become resistant to apoptosis [36, 37] through the activation of phosphatidylinositol 3-kinase (PI3K)/Akt2 signaling [35, 38, 39], and resistance to apoptosis contributes to cancer progression [40–42]. Third, mtDNA depletion in PCa cells induces epithelial-mesenchymal transition [43, 44], which plays an important role in tumor progression [45]. Finally, one study using PCa cell lines showed that reduction in mtDNAcn shifted androgen-dependent PCa cells to a more aggressive androgen-independent phenotype [24].

The findings from our study may have important clinical implications for the management of localized PCa patients. Our results suggested that pre-treatment mtDNAcn in peripheral blood leukocytes could serve as a marker to distinguish between indolent and aggressive PCa. Additionally, our results indicated that low mtDNAcn predicted the presence of aggressive tumors among patients with a negative PSA test (i.e., ≤4 ng/ml). Further, it might predict disease progression independent of commonly used clinical parameters including Gleason score, clinical stage, and PSA. Therefore, mtDNAcn in peripheral blood leukocytes might be included as an independent factor in prediction models to better distinguish between indolent and aggressive PCa and to predict prognosis of PCa patients in order to minimize over-treatment of indolent PCa and under-treatment of aggressive PCa.

Our study has several strengths. It is the largest study to date (our sample size is over 7 times larger than the combined sample size of the three previous studies) to investigate whether mtDNAcn in peripheral blood leukocytes was associated with aggressive features of localized PCa, and the first study to investigate its association with disease progression. We were able to show the associations after adjustment of comprehensive potential confounding factors including age, BMI, smoking, pack-year, clinical and histopathological characteristics of the tumor, and treatments. Consistent and standard protocols were used in the measurements of mtDNAcn, histopathological diagnoses, treatment procedures, and collection of covariates for all of our study patients. Despite these strengths, our study was limited to non-Hispanic white men, and caution should be taken when generalizing the results to other ethnic groups.

In conclusion, the results from this large study of localized PCa patients suggested that low mtDNAcn in peripheral blood leukocytes at diagnosis was associated with aggressive PCa and could further predict disease progression beyond commonly used clinical parameters. Future studies are needed to validate our results in more diverse PCa patient populations.

## MATERIALS AND METHODS

### Study population and data collection

The recruitment and data collection for our study population have been previously reported [46]. Briefly, our study recruited 1,859 non-Hispanic white men with previously untreated localized PCa who were treated at The University of Texas MD Anderson Cancer Center. Among them, measurements of mtDNAcn in peripheral blood leukocytes in blood samples collected at diagnosis were available for 1,751 (94%) patients. At registration, patients filled in questionnaires on demographics, BMI, and smoking history. Study staff conducted detailed chart review of medical records to abstract clinical data including diagnosis date, PSA level at diagnosis, biopsy-proven Gleason score, clinical tumor stage, and treatments. Follow-up information on disease progression was abstracted for patients who received active treatments (*n* = 1,399), and this information was available for 1,266 (90.5%) patients. Follow-up PSA tests were conducted every 3 months for patients with locally advanced tumor (T3a, T3b, and T4) and every 3–6 months for 5 years, then every 6–12 months for 5 years, then annually for patient post definitive therapy. This study was approved by the MD Anderson Cancer Center Institutional Review Board, and written informed consent was obtained from each participant.

### Definition of aggressiveness and disease progression

We defined aggressiveness according to the D'Amico risk criteria [23]: low-risk form of PCa (Gleason score ≤6 and clinical stage T1-T2a and PSA ≤10 ng/ml), intermediate-risk form (Gleason score of 7 and/or stage T2b and/or PSA level >10 and ≤20 ng/mL), and high-risk form (Gleason score ≥8 or clinical stage T2c-T4 or PSA >20 ng/ml). Disease progression was defined as biochemical recurrence (BCR), or presence of local recurrence or distant metastasis, whichever came first, because some cases presented with local recurrence or distant metastasis but no records of a PSA increase. A BCR was defined as a PSA ≥0.2 ng/mL with a second confirmatory level of PSA >0.2 ng/mL for patients treated with radical prostatectomy, as recommended by the American Urological Association Prostate Cancer Guidelines Panel [47], or a PSA rise of 2 ng/mL or more above the nadir PSA for patients who received radiotherapy, according to the Radiation Therapy Oncology Group–American Society for Therapeutic Radiology and Oncology (RTOG-ASTRO) consensus [48].

### Measurements of mtDNA copy number

The relative mtDNAcn in peripheral blood leukocytes was measured by a quantitative reverse transcription-PCR-based method as previously described [49, 50]. In brief, two pairs of primers were used in the two steps of relative quantification of mtDNAcn. One primer pair (ND1-F, 5′-CCCTAAAACCCGCCACATCT-3′; ND1-R, 5′-GAGCGATGGTGAGAGCTAAGGT-3′) was used to amplify the mitochondrial *ND1* gene, and the other primer pair (HGB-1, 5′-GTGCACCTGACTCCTGAGGAGA-3′; HGB-2, 5′-CCTTGATACCAACCTGCCCAG-3′) was used to amplify the single-copy nuclear *HGB* gene.

In the first step, the ratio of the copy number of mitochondrial *ND1* gene to the nuclear *HGB* gene was determined for each sample from standard curves. This ratio is proportional to the mtDNAcn in each cell. The ratio for each sample was then normalized to a calibrator DNA in order to standardize between different runs. The calibrator DNA is from a genomic DNA sample of a healthy control. The PCR mixture of 14 μl contained 1 × SYBR Green PCR Master Mix (Applied Biosystems, Foster City, CA), 215 nM ND1-F and ND1-R (or HGB-1 and HGB-2) primers, and 4 ng of genomic DNA. The thermal cycling conditions for the mitochondrial *ND1* gene amplification were 95°C for 10 min, followed by 40 cycles of 95°C for 15 s and 60°C for 1 min; for the nuclear *HGB* gene amplification, the cycling conditions were 95°C for 10 min, followed by 40 cycles of 95°C for 15 s and 56°C for 1 min. All samples were plated in duplicates on 384-well plates and run on an Applied Biosystems 7900HT Sequence Detection System. The PCRs for *ND1* and *HGB* genes were performed on separate 384-well plates with the same samples in the same well positions to avoid possible position effects.

A standard curve of a serially diluted reference DNA, one negative control and one calibrator DNA were included in each run. For each standard curve, one reference DNA sample was serially diluted 1:2 to produce a seven-point standard curve between 0.3125 and 20 ng of DNA. The *R^2^* for each standard curve was 0.99 or greater. Standard deviations for the cycle of threshold value were acceptable at 0.25. Otherwise, the test was repeated. The intra-assay coefficient of variation for all samples varies from 0.1 to 35% with an overall mean of 5%.

### Statistical analysis

Patients with different host and clinical characteristics at baseline were compared by levels of mtDNAcn using analysis of variance (ANOVA). mtDNAcn was categorized into three groups according to the tertiles in the study population. For the associations between mtDNAcn and aggressiveness of PCa, we calculated odds ratios (ORs) with 95 percent confidence intervals (95% CIs) using multinomial logistic regression with the low-risk PCa as the reference outcome after adjustment of age (<55, 55–65, and > 65 yrs.), BMI (normal/underweight, overweight, and obese), smoking status (never, former, and current smokers), and pack-year (continuous) based on *a priori* knowledge. Patients with missing covariates were not included in the multivariate analyses. In addition, we used restricted cubic spline to model the potential non-linear relationship between mtDNAcn and aggressiveness of PCa; four knots were used and the 99^th^ percentile (2.195) was chose as the reference level.

Time to event for the analysis of disease progression was calculated from the date of diagnosis to the date of disease progression, death, or the latest follow-up, whichever came first. We used Kaplan–Meier survival analysis and log-rank tests to assess the differences in disease progression across different levels of mtDNAcn. We used Cox proportional hazards model to estimate hazard ratios (HRs) and 95% CIs after adjustment of age, BMI, smoking status, pack-year, D’ Amico risk groups (low-risk group, intermediate-risk group, and high-risk group), and primary treatment (radical prostatectomy, radiotherapy, active surveillance or unknown, and other treatments). All statistical analyses were performed using STATA version 10 (StataCorp, College Station, TX, USA). A *P* value < 0.05 (two-sided) was considered statistically significant.

## SUPPLEMENTARY TABLES


