# Supplementary material for: Mitochondrial DNA copy number in peripheral blood leukocytes and the aggressiveness of localized prostate cancer
Source: Oncotarget. 2015 Oct 26;6(39):41988–96. doi: 10.18632/oncotarget.5889 (PMC4747203; doi:10.18632/oncotarget.5889)
Supplement: Supplementary file 1 [file oncotarget-06-41988-s001.pdf]

SUPPLEMENTARY TABLES

Supplementary Table S1: mtDNAcn in peripheral blood leukocytes and aggressiveness of prostate cancer by age, BMI and smoking status

mtDNAcn	Low-risk form of PCa N (%)	Intermediate-risk form of PCa			High-risk form of PCa			*P* for interaction
		N (%)	OR (95% CI)	*P* value	*N* (%)	OR (95% CI)	*P* value	
**Stratified by age**[a]								
Age <60 years								0.98
3rd tertile	119 (42.8)	130 (40.0)	Reference	N/A	27 (27.8)	Reference	N/A	
2nd tertile	102 (36.7)	109 (33.5)	0.92 (0.61–1.41)	0.71	44 (45.4)	1.49 (0.80–2.79)	0.21	
1st tertile	57 (20.5)	86 (26.5)	1.09 (0.68–1.74)	0.73	26 (26.8)	1.58 (0.79–3.19)	0.20	
Age ≥60 years								
3rd tertile	95 (30.5)	155 (30.7)	Reference	N/A	57 (24.3)	Reference	N/A	
2nd tertile	99 (31.8)	160 (31.7)	0.87 (0.57–1.31)	0.50	70 (29.8)	1.28 (0.76–2.18)	0.35	
1st tertile	117 (37.6)	190 (37.6)	1.00 (0.67–1.49)	0.99	108 (46.0)	1.52 (0.92–2.53)	0.10	
**Stratified by BMI**[b]								
BMI <30 kg/m²								0.93
3rd tertile	119 (35.4)	162 (36.7)	Reference	N/A	41 (27.0)	Reference	N/A	
2nd tertile	116 (34.5)	135 (30.5)	0.82 (0.58–1.17)	0.28	52 (34.2)	1.14 (0.68–1.91)	0.61	
1st tertile	101 (30.1)	145 (32.8)	1.00 (0.70–1.45)	0.98	59 (38.8)	1.33 (0.79–2.24)	0.28	
BMI ≥30 kg/m²								
3rd tertile	51 (33.8)	81 (31.9)	Reference	N/A	25 (20.5)	Reference	N/A	
2nd tertile	52 (34.4)	83 (32.7)	1.05 (0.62–1.76)	0.86	47 (38.5)	1.64 (0.85–3.15)	0.14	
1st tertile	48 (31.8)	90 (35.4)	1.21 (0.71–2.05)	0.48	50 (41.0)	1.88 (0.97–3.63)	0.06	
**Stratified by smoking status**[c]								
Never-smokers								0.45
3rd tertile	109 (38.1)	144 (35.2)	Reference	N/A	34 (27.4)	Reference	N/A	
2nd tertile	93 (32.5)	113 (27.6)	0.82 (0.54–1.24)	0.35	40 (32.3)	1.26 (0.69–2.29)	0.45	
1st tertile	84 (29.4)	152 (37.2)	1.21 (0.81–1.82)	0.36	50 (40.3)	1.38 (0.76–2.51)	0.28	
Ever-smokers								
3rd tertile	104 (35.1)	141 (33.6)	Reference	N/A	50 (24.3)	Reference	N/A	
2nd tertile	104 (35.1)	156 (37.1)	0.97 (0.66–1.44)	0.89	74 (35.9)	1.50 (0.90–2.49)	0.12	
1st tertile	88 (29.7)	123 (29.3)	0.88 (0.58–1.33)	0.54	82 (39.8)	1.65 (0.98–2.77)	0.06	

[a]Adjusted for BMI, smoking status and pack-year
[b]Adjusted for age, smoking status and pack-year
[c]Adjusted for age and BMI
Abbreviations: mtDNAcn, mitochondrial DNA copy number; PCa, prostate cancer; OR, odds ratio; CI, confidence interval; BMI, body mass index.

Supplementary Table S2: mtDNAcn in peripheral blood leukocytes and Gleason score of prostate cancer overall and by PSA levels

mtDNAcn	Gleason score ≤6 *N* (%)	Gleason score = 7			Gleason score ≥8		
		N (%)	Adjusted OR[a] (95% CI)	*P* value	*N* (%)	Adjusted OR[a] (95% CI)	*P* value
**Overall**							
3rd tertile	232 (35.9)	295 (33.5)	Reference	N/A	56 (25.2)	Reference	N/A
2nd tertile	223 (34.5)	287 (32.6)	0.96 (0.72–1.27)	0.77	74 (33.3)	1.23 (0.78–1.96)	0.37
1st tertile	192 (29.7)	299 (33.9)	1.11 (0.83–1.48)	0.49	92 (41.4)	1.53 (0.97–2.43)	0.07
**Stratified by PSA levels[b]**							
Among those with PSA ≤4 ng/ml							
3rd tertile	96 (39.2)	60 (32.6)	Reference	N/A	6 (16.2)	Reference	N/A
2nd tertile	84 (34.3)	62 (33.7)	1.10 (0.65–1.86)	0.72	13 (35.1)	3.08 (0.79–11.94)	0.10
1st tertile	65 (26.5)	62 (33.7)	1.35 (0.77–2.35)	0.29	18 (48.6)	4.88 (1.30–18.39)	0.02
Among those with PSA >4 ng/ml							
3rd tertile	136 (33.8)	235 (33.7)	Reference	N/A	50 (27.0)	Reference	N/A
2nd tertile	139 (34.6)	225 (32.3)	0.92 (0.65–1.30)	0.63	61 (33.0)	1.09 (0.65–1.83)	0.75
1st tertile	127 (31.6)	237 (34.0)	1.00 (0.70–1.42)	1.00	74 (40.0)	1.19 (0.71–2.00)	0.51

[a]Adjusted for age, BMI, smoking status and pack-year
[b]*P* for interaction = 0.21
Abbreviations: mtDNAcn, mitochondrial DNA copy number; PSA, prostate-specific antigen; OR, odds ratio; CI, confidence interval.

Supplementary Table S3: mtDNAcn in peripheral blood leukocytes and disease progression among localized prostate cancer patients by age, BMI, smoking status, and D'Amico risk groups

mtDNAcn	Progression *N* (%)	No Progression *N* (%)	Adjusted HR (95% CI)	*P* value	*P* for interaction
**Stratified by age**[a]					
Age <60 years					0.93
3rd tertile (highest)	16 (7.5)	196 (92.5)	Reference	N/A	
2nd and 1st tertile	39 (12.7)	269 (87.3)	1.47 (0.71–3.05)	0.30	
Age ≥60 years					
3rd tertile (highest)	19 (8.5)	205 (91.5)	Reference	N/A	
2nd and 1st tertile	64 (12.3)	458 (87.7)	1.59 (0.82–3.07)	0.17	
**Stratified by BMI**[b]					
BMI <30 kg/m^2					0.44
3rd tertile (highest)	17 (6.9)	228 (93.1)	Reference	N/A	
2nd and 1st tertile	52 (12.3)	370 (87.7)	1.58 (0.87–2.84)	0.13	
BMI ≥30 kg/m2					
3rd tertile (highest)	8 (6.8)	109 (93.2)	Reference	N/A	
2nd and 1st tertile	29 (10.7)	241 (89.3)	1.29 (0.53–3.13)	0.57	
**Stratified by smoking status**[c]					
Non-smokers					0.54
3rd tertile (highest)	15 (6.9)	202 (93.1)	Reference	N/A	
2nd and 1st tertile	41 (10.9)	335 (89.1)	1.74 (0.84–3.61)	0.14	
Ever smokers					
3rd tertile (highest)	20 (9.1)	199 (90.9)	Reference	N/A	
2nd and 1st tertile	60 (13.3)	390 (86.7)	1.40 (0.77–2.56)	0.27	
**Stratified by D'Amico risk group**[d]					
Low/intermediate-risk form of PCa					0.92
3rd tertile (highest)	21 (5.7)	349 (94.3)	Reference	N/A	
2nd and 1st tertile	44 (6.9)	596 (93.1)	1.48 (0.75–2.92)	0.26	
High-risk form of PCa					
3rd tertile (highest)	14 (21.2)	52 (78.8)	Reference	N/A	
2nd and 1st tertile	59 (31.1)	131 (68.9)	1.58 (0.77–3.23)	0.21	

[a]Adjusted for BMI, smoking status, pack-year, D'Amico risk groups, and and primary treatment
[b]Adjusted for age, smoking status, pack-year, D'Amico risk groups, and and primary treatment
[c]Adjusted for age, BMI, D'Amico risk groups, and and primary treatment
[d]Adjusted for age, BMI, smoking status, pack-year, and primary treatment
Abbreviations: mtDNAcn, mitochondrial DNA copy number; BMI, body mass index; HR, hazard ratio; CI, confidence interval.
